# Multi-layered core-sheath fiber membranes for controlled drug release in the local treatment of brain tumor

**DOI:** 10.1038/s41598-019-54283-y

**Published:** 2019-11-29

**Authors:** Daewoo Han, Riccardo Serra, Noah Gorelick, Umailla Fatima, Charles G. Eberhart, Henry Brem, Betty Tyler, Andrew J. Steckl

**Affiliations:** 1 Nanoelectronics Laboratory, Department of Electrical Engineering and Computer Science, University of Cincinnati, Cincinnati, OH USA; 2 Department of Neurosurgery, Johns Hopkins University School of Medicine, Baltimore, MD USA; 3 Departments of Pathology, Oncology, and Ophthalmology, Johns Hopkins University School of Medicine, Baltimore, MD USA; 4 Departments of Biomedical Engineering, Oncology, and Ophthalmology, Johns Hopkins University School of Medicine, Baltimore, MD USA

**Keywords:** Surgical oncology, Biomaterials

## Abstract

Interstitial chemotherapy plays a pivotal role in the treatment of glioblastoma multiforme (GBM), an aggressive form of primary brain cancer, by enhancing drug biodistribution to the tumor and avoiding systemic toxicities. The use of new polymer structures that extend the release of cytotoxic agents may therefore increase survival and prevent recurrence. A novel core-sheath fiber loaded with the drug carmustine (BCNU) was evaluated in an *in vivo* brain tumor model. Three-dimensional discs were formed from coaxially electrospun fiber membranes and *in vitro* BCNU release kinetics were measured. *In vivo* survival was assessed following implantation of discs made of compressed core-sheath fibers (NanoMesh) either concurrently with or five days after intracranial implantation of 9L gliosarcoma. Co-implantation of NanoMesh and 9L gliosarcoma resulted in statistically significant long-term survival (>150 days). Empty control NanoMesh confirmed the safety of these novel implants. Similarly, Day 5 studies showed significant median, overall, and long-term survival rates, suggesting optimal control of tumor growth, confirmed with histological and immunohistochemical analyses. Local chemotherapy by means of biodegradable NanoMesh implants is a new treatment paradigm for the treatment for brain tumors. Drug delivery with coaxial core-sheath structures benefits from high drug loading, controlled long-term release kinetics, and slow polymer degradation. This represents a promising evolution for the current treatment of GBM.

## Introduction

Delivering therapeutics to the targeted site for a long-term period and in a controlled manner is one of most important weapons in the battle against various diseases. For malignant cancers, the long-term local delivery of anti-cancer therapeutics using advanced materials and their various structures is being intensively investigated. In particular, the use of electrospun fibrous membranes has emerged recently as an attractive and versatile approach, as illustrated in a review of its early stage of development by Chen *et al*.^1^.

Glioblastoma multiforme (GBM), the most common and aggressive primary brain cancer, has an incidence of approximately 3.2 cases/100,000 persons/year, with a 5-year survival rate of 5.1%, a marked resistance to multimodal chemo- and radio-therapy (RT), and had a mean survival time of less than 15 months^2,3^. Classified in 2016 by the WHO as a Grade IV glial tumor^2^, GBM treatment relied mainly on the combination of maximally safe surgical resection followed by radiation therapy (RT) and concomitant chemotherapy with temozolomide (TMZ - Stupp Protocol)^4^. One of the main obstacles to effective drug delivery is posed by the blood-brain barrier (BBB), an anatomical and functional barrier to solute and chemotherapy diffusion to the brain and tumor parenchyma^5^. To overcome this limitation, local delivery of therapeutics to the central nervous system (CNS) has been investigated by placing biocompatible and biodegradable polymeric wafers inside the tumor resection cavity. This strategy led to the development and clinical translation of Gliadel^®^, a carmustine (bis-chloroethyl-nitrosourea (BCNU))-impregnated polyanhydride poly-(1,3 bis[p-carboxyphenoxy] propane-co-sebacic acid) 20:80 polymeric (p(CPP):SA) wafer that can be positioned in contact with the resection walls and paired with conventional RT and systemic TMZ administration^6–9^. This triple therapy approach has increased the median survival for patients with GBM to nearly 21 months and in multiple retrospective studies and meta-analyses has demonstrated a consistent increase in median survival for both patients with newly diagnosed and recurrent GBMs^7,10–12^. The main limitations of this approach consist of rapid BCNU hydrolysis, fast polymer degradation, and short-term drug release kinetics. The optimization of these issues, paired with more effective chemotherapeutics could further improve the median survival rate and quality of life of these patients.

**Figure 1:**
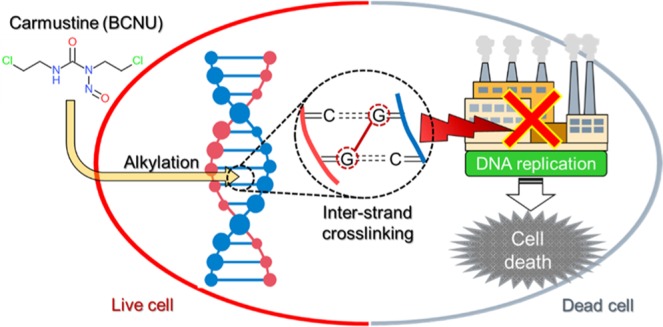
Illustration of the mechanism of action of the alkylating agent BCNU on a tumor cell.

BCNU, a nitrogen mustard β-chloro-nitrosourea irreversibly binds to DNA by forming inter-strand crosslinks, as illustrated in Fig. 1. This prevents strand separation and DNA replication, resulting in cell apoptosis. Historically, BCNU has played a pivotal role in GBM treatment, first as a systemic chemotherapy, and more recently, over the last 20 years, as a locally administered compound from the Gliadel^®^ wafer.

Electrospun nanofiber membranes provide controlled and sustained drug release over long periods of time. The electrospinning technique enables the synthesis of continuous nanofibers, with diameters ranging from tens of nanometers to microns and many meters in length. Fiber electrospinning and its many applications have been established and demonstrated^13^. The physical and chemical properties of the nanofibers can be manipulated by controlling polymer concentration, additives, and solvent selection. Moreover, the versatility of the electrospinning technique can be further extended by forming core-sheath fibers using two fluid coaxial nozzle electrospinning^14^. In addition to the basic advantages of fiber electrospinning (such as controllability of fiber morphologies and compositions, extremely high surface area, and porous structure) coaxial electrospinning enables: (a) combination of two different sets of properties from each polymer into one fiber; (b) encapsulation of multiple functional molecules into the specific layer; and (c) control of release rate by the design of the fiber structure and compositions. The final and critical step for producing a practical delivery mechanism is the formation of an easy-to-handle and robust (pill-like) disc from densified multi-layer fiber membranes. This approach increases the loading capacity of the incorporated drug per unit volume, lowers the initial burst release, and enhances the sustainability of drug release.

Over the past years, different anti-cancer drugs have been incorporated into electrospun fibers and tested in cell and animal models. Xie *et al*. showed the efficacy of paclitaxel-loaded homogeneous poly(lactic-co-glycolic acid) (PLGA) fibers in treating *in vitro* C6 rat glioma, demonstrating sustained drug release for up to 2 months^15^. Similarly, Xu *et al*. incorporated BCNU in polyethylene glycol – poly(L-lactic acid)(PEG-PLLA) fibers and measured the cytotoxic activity in C6 cells over 72 hours^16^. Coaxially electrospun core-sheath fibers containing paclitaxel in the core with poly(L-lactic acid-*co*-*ε*-caprolactone) (P(LLA-CL)) sheath effectively inhibited the activity of HeLa cells *in vitro*, but experienced significant initial burst release within 24 hr^17^. In 2013, BCNU-loaded poly[(D,L)-lactide-*co*-glycolide] (PLGA) single fibers^18^ show sustained release of BCNU up to 6 weeks in the cerebral cavity of rats with no obvious inflammation of brain tissues. More recently in 2017, fluorouracil and paclitaxel encapsulated PCL core-sheath fibers demonstrated *in vitro* cancer cell viability lower than that of the pristine drugs^19^.

The drug-polymer electrospun fibers for GBM treatment indicated above do not match current state-of-art wafers in release kinetics and *in vivo* survival. All previous studies with electrospun membranes for brain cancers used C6 glioma cells for both *in vitro* and *in vivo* experiments. In contrast, the 9L gliosarcoma cell line used for our animal studies has been widely used without potential alloimmune response, providing important information related to brain tumor biology and therapy^20^. Our animal studies using the 9L gliosarcoma should provide reliable and unique information to evaluate the effect of electrospun membranes. Another issue in previously reported fibers formed by conventional single fluid electrospinning are resulting drug release profiles strongly affected by high drug solubility (and rapid release) in aqueous media. In our approach, the effect of drug solubility from discs made of core-sheath fibers can be minimized by encapsulating the drugs within the core with a hydrophobic sheath layer. This benefit is significant when targeted to long-term release with hydrophilic drugs. Core-sheath fiber membranes also provide higher drug encapsulation efficiency and better protection and stability under various environmental conditions. The multi-layered membrane discs made of coaxial fibers reported here can in fact provide long-term release kinetics regardless of the nature of the encapsulated drug and could be a promising alternative to polymeric bulk wafers.

**Figure 2:**
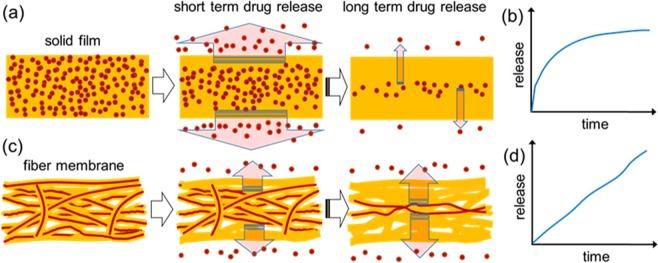
Comparison of drug release from (**a**) solid film and (**c**) multi-layered fiber membrane wafer, and corresponding exemplary release kinetics over time for (**b**) solid-film and (**d**) multi-layered fiber membrane.

The concept for consistent long-term delivery without diffusion-limited saturation is illustrated in Fig. 2. For the carrier consisting of a conventional thick solid disc (Fig. 2a), drug molecules diffuse out from the surface. Therefore, drugs near surfaces are quickly released at the start, then the release rate decreases as the diffusion length becomes longer, as illustrated in Fig. 2b. On the other hand, the multi-layered porous fiber membrane discs (Fig. 2c) provide fairly uniform diffusion lengths over time (Fig. 2d), because aqueous media gradually wets the disc from the outside due to the hydrophobic nature of fiber surfaces. Therefore, consistent long-term release of encapsulated drugs with minimized initial burst release is obtained. Once the membrane is totally hydrated, the remaining drug molecules are released with a relatively slower rate.

In this study we demonstrated that local administration of BCNU-impregnated NanoMesh obtained from densified multi-layer fiber membranes is capable of achieving improved median and overall survival in F344 rats implanted with intracranial 9L gliosarcoma as compared to untreated controls. Furthermore, the fiber membranes were both biocompatible and non-toxic during brain implantation for longer than 150 days, opening a promising avenue of research for future developments of this novel technology.

## Results

### Fiber morphology and disc transformation

**Figure 3:**
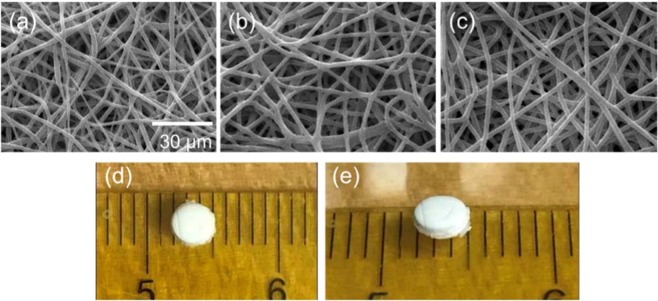
SEM images of NanoMesh: (**a**) control samples without core material; (**b**) thin sheath BCNU encapsulated fibers (UC-1, 36 layers); (**c**) thick sheath BCNU encapsulated fibers (UC-2, 18 layers). Photographs of NanoMesh (UC-1) (**d**) top view and (**e**) angled view.

Membranes with core-sheath fibers encapsulating BCNU in the pCPP-SA core and with PCL sheath were successfully produced with different sheath thicknesses using coaxial electrospinning. As shown in scanning electron microscope (SEM) images (Fig. 3a–c), uniform fiber formation was obtained with an average fiber diameter of 1.9 ± 0.5 µm (control without core material), 2.2 ± 0.2 µm (core-sheath fiber with thin sheath ~0.36 µm), and 2.4 ± 0.2 µm (core-sheath fiber with thick sheath ~0.56 µm). For the purpose of intracranial implantation, the drug delivery platform used was in the form of a wafer to provide easy handling during the surgical procedure and maintain mechanical integrity after implantation. Electrospinning generally produces very thin fabric-like membranes with high porosity. Although this highly porous network is a very attractive feature for many applications (such as tissue scaffolds^21^, and sensors^22^) it can limit the drug loading density within a specific volume because almost 80% of volume is a void space. In our previous immunoassay research using electrospun membranes, the “fold-and-press” process^23^ was utilized to densify the membrane to obtain an amplified fluorescence signal while maintaining the benefit of high surface area of the nanofiber network. This approach was adopted in the current work on drug release. Coaxial fiber membranes folded multiple times provided the starting material from which robust discs (NanoMesh) were “punched” out. An example of a multi-layered (~36 layers) membrane disc is shown in Fig. 3d,e. Prepared membranes were folded into either 36 (UC-1, thin sheath) or 18 (UC-2, thick sheath) layers and punched into 3 mm (diameter) × 1 mm (thickness) discs, weighing ~7–9 mg. Due to their respective sheath thickness, UC-2 (thick sheath) has fewer layers than UC-1 (thin sheath) in order to maintain similar disc thickness of ~1 mm. The NanoMesh size of 3 mm in diameter and 1 mm in thickness is determined by the need to implant into small rat brains. For eventual human use, the size and the shape of the NanoMesh can be easily adjusted. This approach represents a very simple and versatile method to fabricate the drug incorporated disc type nanofiber membranes.

**Figure 4:**
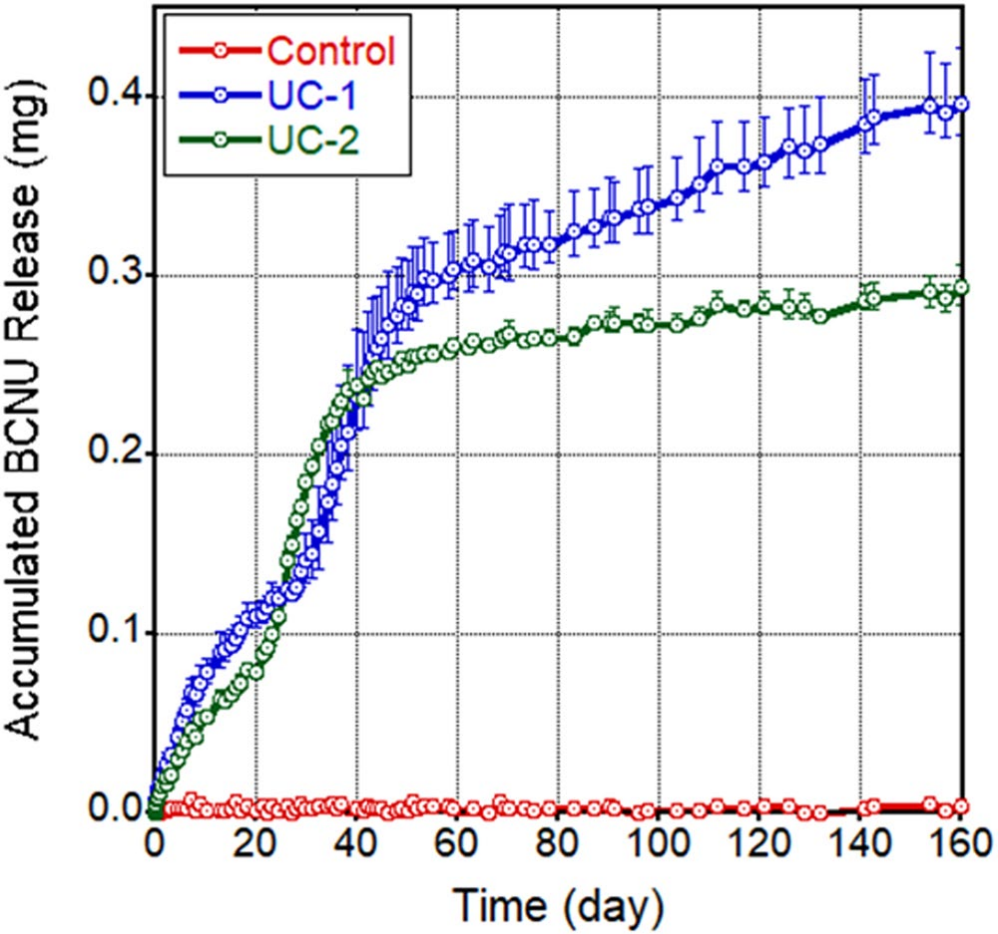
Drug release profile of NanoMesh discs in Trial 1: UC-1 (blue), 36-layer BCNU/pCPP-SA core & PCL sheath; UC-2 (green) 18-layer BCNU/pCPP-SA core & PCL sheath; Control (red), No core & PCL sheath. (n = 3) Detailed compositions are shown in Table 1.

As shown in Fig. 4, no initial burst release of BCNU was observed from the NanoMesh disc and continuous release was observed up to 160 days. A low initial release rate was observed followed by an acceleration starting at Day 30 and then a reduction in slope at Day 50 continuing on until Day 160, the end of measurements. The varied release rate may possibly be due to the multi-layered structure of the NanoMesh discs. Initially, only the disc surface is wetted and can therefore release drugs, thus minimizing burst release. As the aqueous media gradually wets inner layers of the disc (~Day 30), there is an increasingly larger number of fibers that take part in the release process, leading to an acceleration of the release rate. Finally, when the entire disc is fully wetted, the remaining drugs in the fiber core are released at a constant, slower rate that results in long-term release.

**Figure 5:**
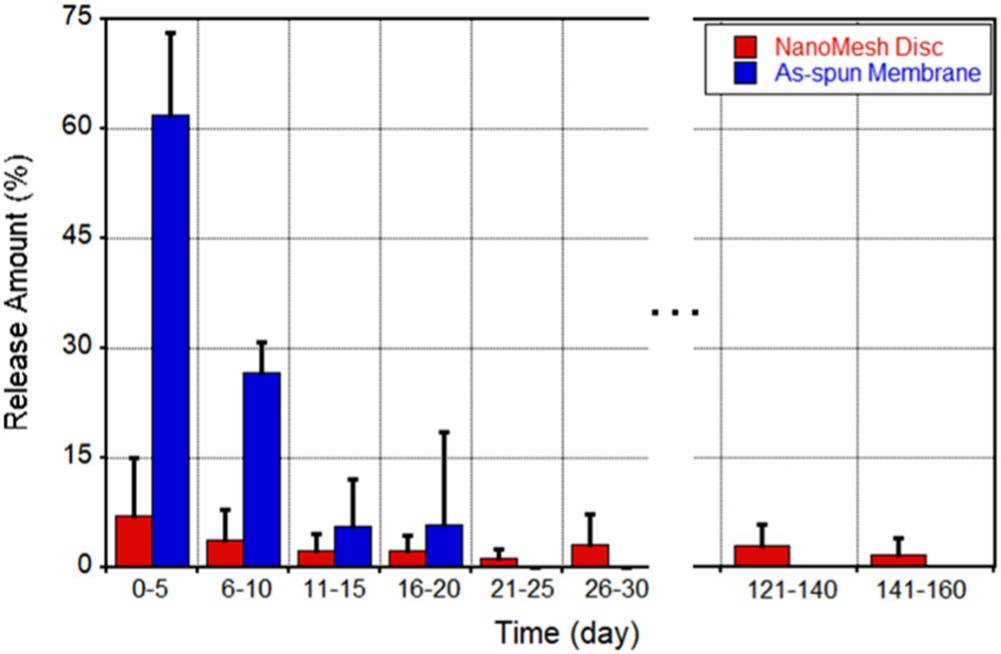
Comparison of drug release profile from as-electrospun thin fiber membrane (blue) and NanoMesh disc (red) over a 160-day period (n = 3).

Drug release kinetics from different forms of nanofibers are compared in Fig. 5. As-electrospun thin membranes and transformed UC-1 NanoMesh samples were prepared with the same core-sheath fiber/drug composition and amount. Thin membrane samples released significant amount of incorporated BCNU (~62%) within the first 5 days and no further release was noticed after 20 days. However, the NanoMesh samples displayed much lower release ~7% in the initial 5 days followed by consistent release up to 160 days. This long-term release was due to the hydrophobic barrier that covers the entire disc producing gradual wetting, as discussed in Fig. 2. In contrast, the thin membranes were quickly hydrated because of the high porosity of membrane and its low thickness, leading to a relatively short-release timespan compared to the fiber disc sample. Transforming thin membranes into discs not only increases the drug loading capacity but also lengthens the drug delivery period significantly, resulting in a very attractive local drug delivery vehicle.

### Local chemotherapy with NanoMesh improves survival ***in vivo*** in three separate trials

BCNU-impregnated UC-1 (~7.9% BCNU) and UC-2 (~5.1% BCNU) NanoMesh discs were investigated in F344 rats implanted with 9L gliosarcoma in several trials that assessed safety, toxicity, membrane degradation, and efficacy. A total of 94 F344 rats were included in the studies and followed for 150 days.

#### Trial 1

**Figure 6:**
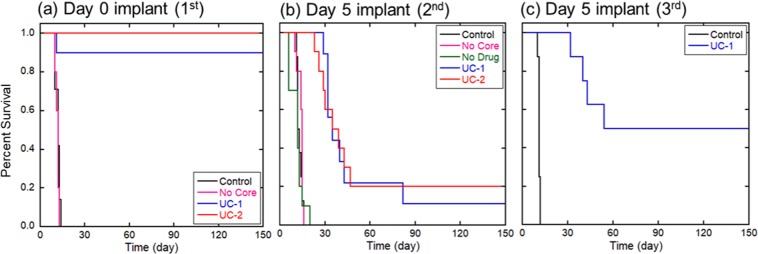
Intracranially implanted NanoMesh significantly prolong survival in 9L intracranial gliosarcoma: (**a**) Trial 1, Day 0 implant, Implanted UC-1 and UC-2 NanoMeshes resulted in 86% and 100% LTS, respectively; (**b**) Trial 2, Day 5 implant, a significant increase in median survival of 36 and 37 days for UC-1 and UC-2 treated groups, respectively, (p < 0.0001 vs. control) was observed; (**c**) Trial 3, Day 5 implant, confirmed significant increase in median survival in UC-1 treated animals with 50% LTS (p < 0.0001 vs. control).

Trial 1 consisted of intracranial gliosarcoma placement followed by implantation on Day 0 of either “Control” (untreated) (n = 7), “No Core” (NanoMesh with no core and no drug) (n = 5), UC-1 NanoMesh (n = 7) or UC-2 NanoMesh (n = 6) in a cohort of 31 animals. Untreated control rats and rats that were implanted with the “No Core” NanoMesh had median survivals of 12 days (Fig. 6a). Six of the 7 animals (85.7%) implanted with the UC-1 NanoMesh and 100% of the UC-2 NanoMesh implanted rats lived until the conclusion of the study on Day 150 and were deemed long-term survivors (LTS) (p = 0.0027 and 0.0004, for untreated control vs. UC-1 and UC-2, respectively). No animals showed signs of neurologic compromise or systemic illness due to the implant throughout the duration of the experiment.

Three additional animals were implanted with tumor and either the UC-1 or the UC-2 NanoMesh. These animals were euthanized at prefixed time points (1 rat/group on day 40, 2 rats/group on day 80). Brains were perfused and histological analyses showed no macroscopic tumor growth on necropsy. The median survival of treated rats with NanoMesh was significantly increased as compared to the control groups, representing a very significant potential for the GBM treatment.

#### Trial 2

To more closely mimic the clinical situation, we assessed the efficacy of BCNU-impregnated UC-1 and UC-2 NanoMeshes in F344 rats with established intracranial 9L gliosarcoma, investigating median and long-term survival of animals treated 5 days after tumor implantation. For Trials 2 and 3 animals were inoculated with tumor pieces that were allowed to grow intracranially for 5 days before disc implantation. During this 5-day period, this aggressive tumor undergoes exponential growth intracranially as well as invasive growth into the normal brain tissue. This established tumor model mimics the clinical situation in which a large tumor mass has led to clinical symptoms. The survival rates of the groups in Trial 2 are seen in Fig. 6b. F344 rats that received intracranial 9L gliosarcoma were randomly divided into the following groups: Untreated control (n = 8); No Core NanoMesh (n = 10); No Drug NanoMesh (n = 10); UC-1 NanoMesh (n = 10); UC-2 NanoMesh (n = 9). Animals were again followed daily for 150 days. While the untreated controls had a median survival of 12 days, the No Core and the No Drug NanoMesh groups showed no significant difference from untreated controls with median survivals of 15 and 12 days, respectively (Fig. 6b). The two groups that received BCNU-loaded UC-1 or UC-2 NanoMesh had median survivals of 35 and 37 days, respectively, demonstrating a statistically significant increase for both as compared to all control groups (p < 0.0001). One UC-1 (11% of the total) and two UC-2 (20%) animals survived until day 150 and were deemed LTS. Upon histological examination no tumor growth was found and no signs of systemic or neurological toxicity was observed in any of the animals. One healthy rat from the “No Drug” group was euthanized on Day 14 to examine for any possible histological changes due to the NanoMesh alone (no drug). Other than slight reactive gliosis and a localized inflammatory response the animal was healthy and showed no deleterious effect. (Supplemental Fig. S1).

#### Trial 3

**Figure 7:**
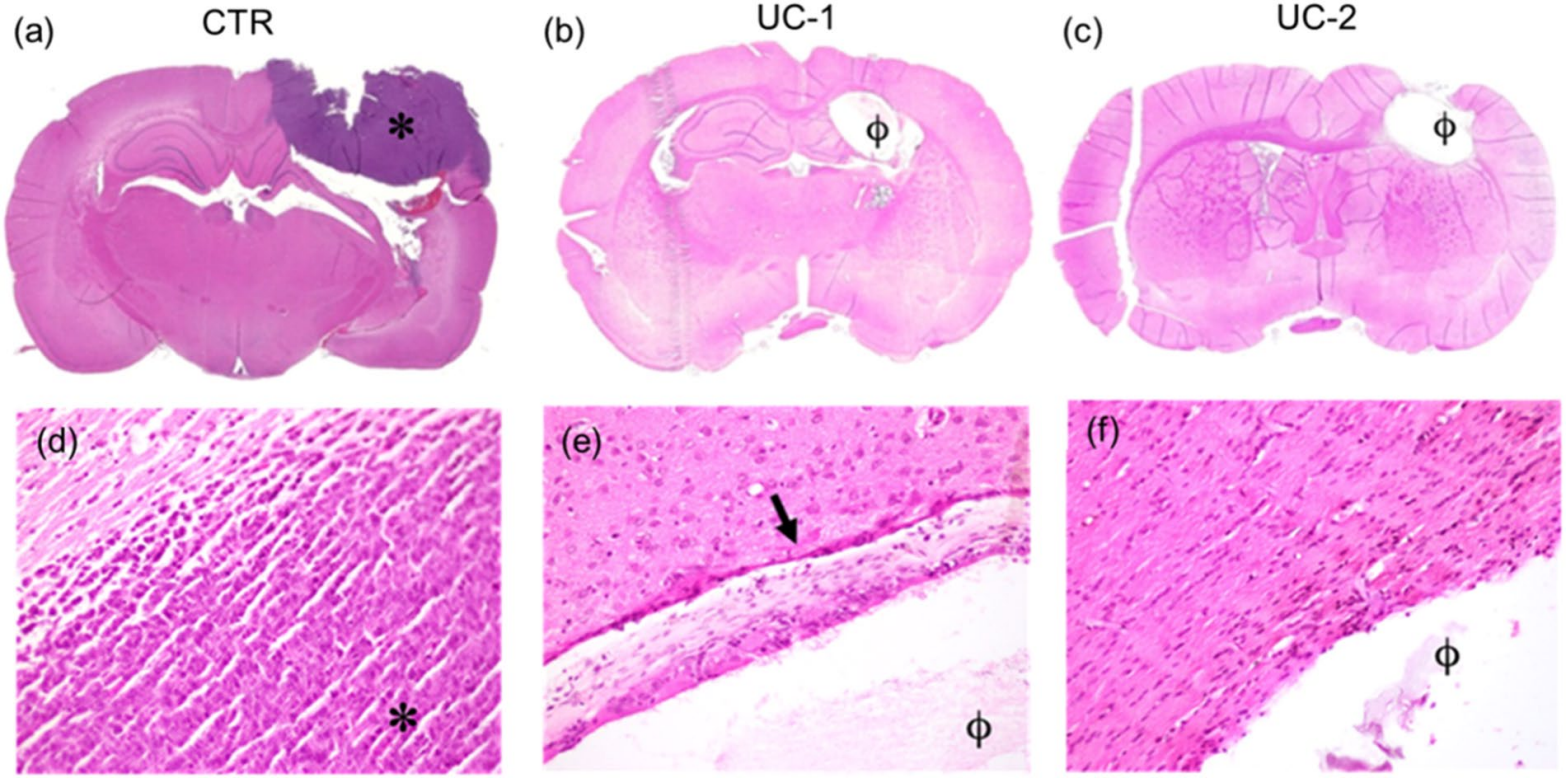
Trial 1 Histology (Day 0 Treatment). Coronal brain sections (top row, 2.5×) demonstrate presence of gross tumor (*) in **(a)** Untreated Control (CTR) rat, and polymer remnants (ϕ) with no evidence of residual bulk tumor in rats treated with **(b)** UC-1 and **(c)** UC-2 NanoMeshes. High magnification (20×) images (bottom row) demonstrate hypercellular tumor with some infiltration at the margin of **(d)** control brain, and few reactive cells (arrow) in **(e)** UC-1- and **(f)** UC-2-treated brains. Control rats died spontaneously on day 10 from 9L gliosarcoma, while treated animals were healthy long-term survivors sacrificed at day 150 for histological analysis.

**Figure 8:**
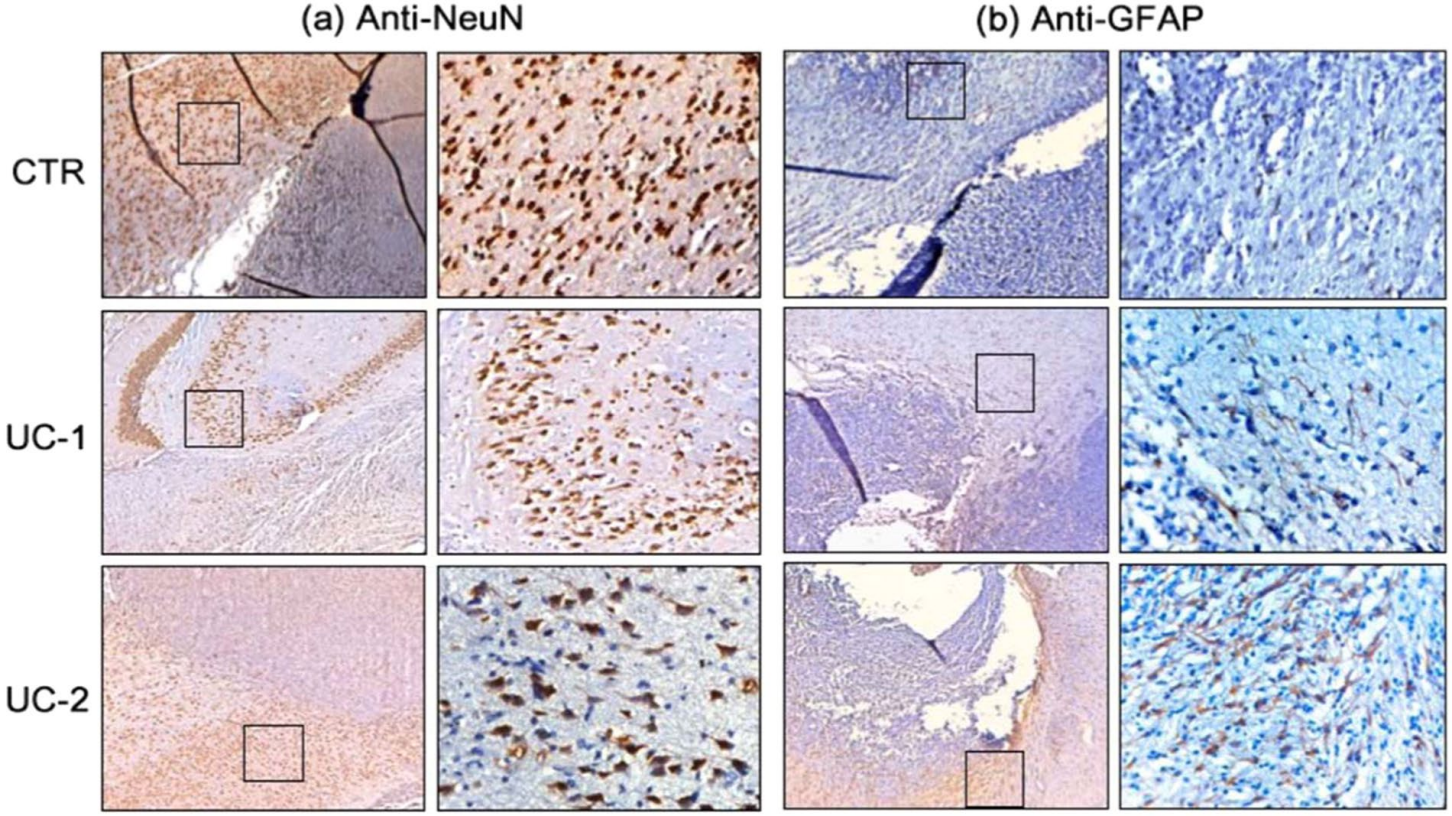
Trial 2 Immunohistochemistry (Day 5 Treatment). IHC staining of brain samples from Untreated Control (CTR) (top row), UC-1- (middle row), and UC-2-treated rats (bottom row) that died spontaneously from 9L gliosarcoma at days 12, 43, and 30, respectively. For each pair of images, left is 2.5× magnification of the area at the margin between tumor and healthy tissue and right is 20× magnification of healthy tissue in proximity to tumor margin (general location demonstrated by square boxes in 2.5× photos). Anti-NeuN **(a)** and anti-GFAP (**b)** staining demonstrates no qualitative decrease in signal from healthy neurons and astrocytes among treated brains compared to control. Images of astrocytes and neurons were obtained from hippocampal and cortical areas, adjacent to the tumor margin near wafer implant.

This study was to confirm the findings from Trial 2 in an established tumor model and compared UC-1 NanoMesh (n = 8) with untreated controls (n = 8). Since UC-1 showed statistically comparable efficacy to UC-2 in both previous trials, UC-1, which had the higher loading dose of BCNU (~7.9% BCNU), was chosen to gain additional evidence regarding the efficacy of this formulation. Untreated controls had a median survival of 11 days compared to the UC-1 group that did not reach median survival and resulted on 50% LTS. (p < 0.0001) (Fig. 6c). In addition, 4 of the UC-1 treated animals (50%) were LTS and survived until they were sacrificed on Day 150 with no signs of toxicity or tumor growth, as evidenced by lack of symptoms of neurological deterioration and maintenance of healthy weights in all LTS animals. Furthermore, histology (Figs. 7, 8) shows no evidence of particular toxicity to astrocytes and neurons in animals with wafers, compared to untreated controls.

No significant differences in survival or behavior were observed between animals that received UC-1 (~7.9% BCNU) and UC-2 (~5.2% BCNU) NanoMeshes during these studies. This similarity is possibly related to their comparable BCNU release rates shown in Fig. 4 during the first 60 days. At longer periods an increasing difference in released BCNU amount is observed due to the difference of incorporated BCNU amounts (UC-1 > UC-2). However, this difference in released amount of BCNU does not seem to affect the long-term animal survivability. Ultimately, the lowest dose sufficient for obtaining long term survival is desired.

### Histopathological analysis

Histological analysis of brain slides was performed by an independent and blinded neuropathologist, and confirmed the presence of a tumor mass in the left hemisphere for all controls, demonstrated by the presence of a large hypercellular region with infiltration at the margins (Fig. 7a,d). The lesion volume was larger in untreated control animals as well as those animals implanted with No Core membranes (Supplemental Fig. S2). These control groups had deep brain structure invasion and distortion and high cellularity. Brains harvested from asymptomatic UC-1- and UC-2-implanted rats showed absence of gross malignant tumor growth (Figs. 7b,c). Close histological analysis of the margins that surrounded residual polymer demonstrated mild-to-moderate gliosis with few reactive cells in the regions around the membranes (Figs. 7e,f).

Brains of representative animals from Trial 2 were fixed and examined in order to assess cytotoxicity within the microenvironment surrounding the tumor/membrane implantation site. BCNU-loaded membranes showed a diffuse inflammatory response surrounding the implant, and the degree of chronic inflammation gradually improved over time, as shown by the decrease in reactive cells around the residual polymeric implants at day 150 (Supplemental Fig. S3). This indicates that the NanoMesh disc is not causing toxicities in the lesion cavity during the long-term implantation period. Immunohistochemical staining against neuronal and astrocytic markers (NeuN, GFAP) in brains of rats that died spontaneously from tumor showed no qualitative decrease in signal among the brains treated with BCNU-containing membranes in comparison to the untreated controls, indicating further evidence that direct toxicity of the membranes was not a major factor in their deaths (Fig. 8).

### Post-implant electron microscopy analysis of nanomesh discs

**Figure 9:**
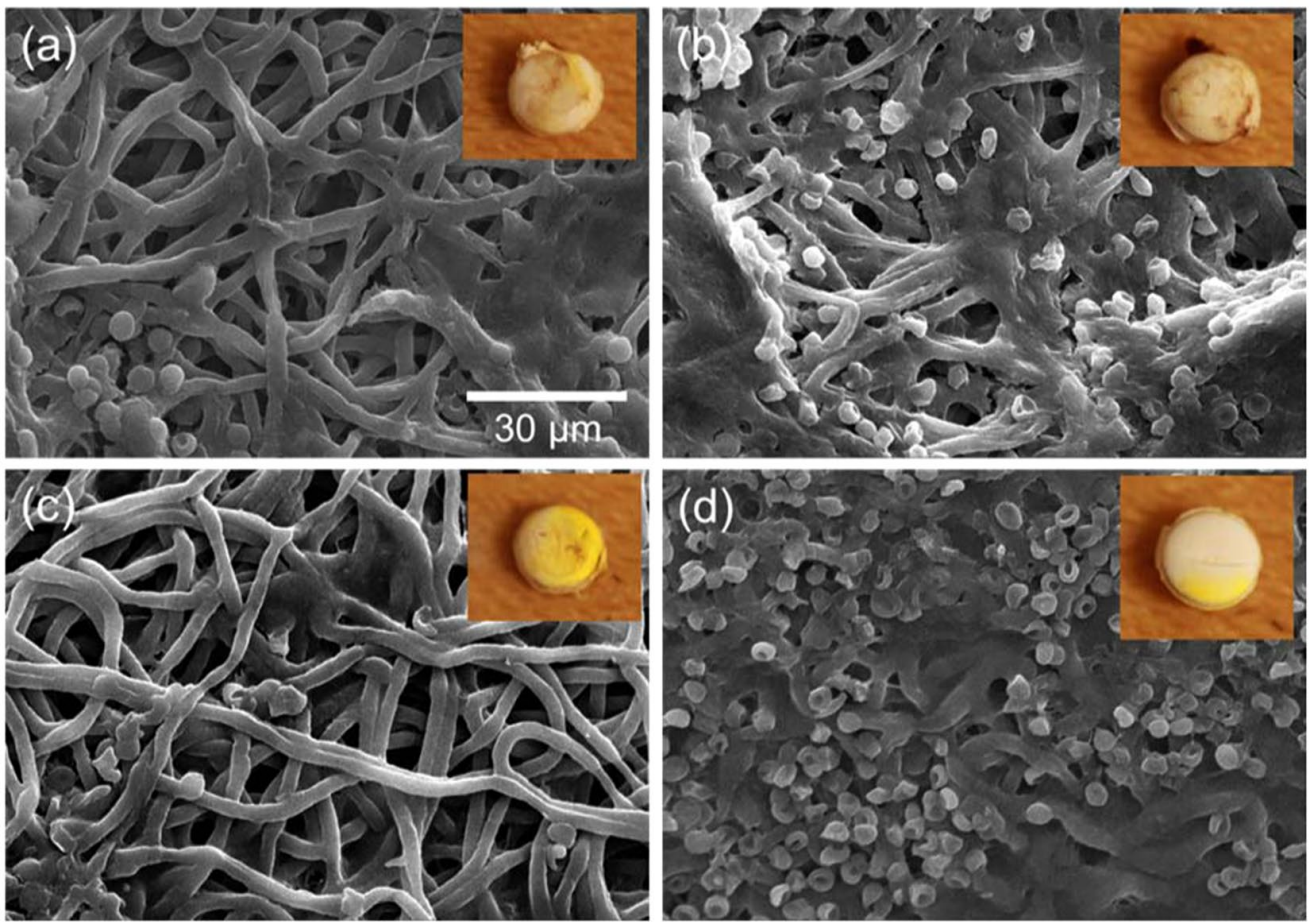
SEM images of post-implantation NanoMesh discs from Trial 2 samples: (**a**) “No Core” at 18 days post-implantation; (**b**) “No Drug” at 12 days post-implantation; (**c**) UC-1 at 32 days post-implantation; (**d**) UC-2 at 34 days post-implantation; insets are images of corresponding discs.

Interestingly, implanted NanoMesh discs remained intact after many days of intracranial implantation, which indicates its suitability for long term local therapy use. SEM images of “post-implantation” membranes recovered following *in vivo* rat experiments (Fig. 9a–d) clearly showed that the porous non-woven microstructure and fiber morphologies were still intact, although fibers were slightly flattened and densely packed, most likely due to the pressure from densifying multi-layered membranes into discs. The inner layers of the post-implant membranes (Fig. S4 in Supplementary Information) showed similar morphologies and dense fiber network without any cellular components. Cells were not visualized penetrating the implanted discs, preventing any clogging inside the discs. This should enable the long-term release of drugs from the Nano-Mesh disc.

## Discussion

The treatment of GBM, one of the most common and aggressive human brain malignancies, has benefitted over the last decades from an innovative drug delivery strategy. Local administration of therapeutics to the brain has led to a significant improvement in survival rates^7,10–12^, and a remarkable number of technologies that use chemotherapy have been investigated and developed in recent years^24–27^.

One of the limitations of drug-impregnated polymeric wafers may be due to the burst release of the loaded cytotoxic agent, with only a modest fraction of drug being delivered weeks after wafer implantation. To address this pressing need we investigated the use of a novel multi-layered membrane disc composed of compressed coaxial electrospun fibers (NanoMesh), loaded with different w/w percentages of BCNU, and implanted in an orthotopic rat model of gliosarcoma.

This electrospun membrane-based technology, given its favorable release kinetics and ability to incorporate hydrophilic compounds, could be an interesting candidate matrix formulation for the delivery of novel molecules for the treatment of GBM and other brain malignancies. Here, we show preclinical proof of concept of a novel compound for safe local delivery of BCNU by means of compressed electrospun membranes.

Transforming the electrospun membranes into the NanoMesh disc form increases drug loading capacity by reducing membrane porosity while maintaining fibrous structures. Considering material density, core/sheath flow rate ratio, and disc/membrane dimensions, the porosity of UC-1 and UC-2 discs ranged from ~2 to 5%, while the porosity of the membrane was ~82%. The porosity was significantly reduced by applied pressure during transformation. Similarly, based on disc/membrane weights and dimensions, the drug loading capacity of UC-1 NanoMesh discs was calculated to be ~95 µg/mm^3^, while the drug loading capacity of UC-1 membrane was ~17 µg/mm^3^. Clearly, the drug loading capacity was increased by ~5.6 times after transforming into the disc type. Excellent long-term release kinetics without noticeable initial burst release is also obtained by gradual NanoMesh hydration from the outer layers in conjunction with core-sheath fibers with hydrophobic sheath layer minimizing the effect of drug solubility on release kinetics. Significant initial release is expected from NanoMesh membranes  consisting of single fibers loaded with hydrophilic drugs. Of significant added potential further research can be pursued to mimic the current systemic ‘cocktail’ chemotherapy with multiple anti-cancer drugs. Because the NanoMesh is hydrated from outer layers to inner layers, different anti-cancer drugs can be incorporated into different layers within the NanoMesh to be released either in concurrent or sequential manner. This may also assist in preventing drug resistance, as well as providing a synergistic effect from different types of anti-cancer drugs. Controlled ‘cocktail’ local therapy may bring a new paradigm to the GBM therapy.

*In vivo* studies demonstrated safety of the NanoMesh membranes in rodents, including No Core, and No Drug NanoMeshes, as well as the BCNU-loaded NanoMeshes. In these combined studies of nearly 100 animals there were no early deaths attributable to wafer implantation alone. The histopathological analysis confirmed a modest inflammatory infiltrate in healthy rats euthanized on Day 40 with improvement of this reaction in brain samples collected on Day 150 (Supplemental Fig. S3). Further analysis of samples from animals treated with the same wafers (UC-1 and UC-2) and sacrificed on Days 80 and 150 demonstrated clearance of the inflammatory process and only a modest gliosis remaining. The evidence of inflammation in conjunction with the improvement in survival in these groups supports the hypothesis of certain types of inflammation potentiating an antitumor immune response. Mathios *et al*. demonstrated that local chemotherapy, given by a BCNU wafer, enhanced systemically delivered^28^ immunotherapy by destroying the tumor microenvironment and attracting immune cells to the tumor area, whereas systemic chemotherapy alone displayed a non-specific toxicity toward cancer cells and the immune system.

In addition to the NanoMesh membrane’s intracranial safety profile, efficacy was demonstrated in rodents co-implanted with 9L tumor and BCNU-loaded membranes leading to 85% and 100% LTS in the groups implanted with UC-1 and UC-2, respectively. This significant improvement in median survival was also confirmed in a more clinically relevant established tumor model with wafer implantation five days after tumor placement. The aggressive growth of 9L gliosarcoma was demonstrated by the short median survival of 12 days in the control group, creating a challenging test for novel therapies. Nonetheless, animals treated with UC-1 and UC-2 NanoMesh 5 days after tumor implantation achieved a statistically significant increase in median survival compared to all control groups, confirming the trend observed during Trial 1 and providing further proof of effective and prolonged intracranial BCNU release. A third efficacy study with UC-1 NanoMesh discs implanted five days after tumors maintained a 50% survival rate after 150 days in the treatment arm, compared to a median survival of 11 days in the untreated control group, further demonstrating the reproducibility and efficacy of this membrane fiber.

Notably, in all brains harvested from animals that died spontaneously or were euthanized at prefixed time points it was possible to visualize and extract a considerable amount of the original NanoMesh discs, showing different degrees of erosion with substantial preservation over periods of up to 150 days from the initial implantation (see Figs. 9 and S4). This is due to the very slow degradation rate of PCL material. Although no toxicity was observed in rat brain during *in-vivo* tests, faster degradation rate can be acquired by replacing PCL with other biocompatible hydrophobic polymers such as PLGA. Based on *in vitro* data shown in Figs. 4 and 5, because accumulated release at 150 days was ~3.6 O.D. (0.4 mg) compared to the accumulated release from the thin membrane of ~6.2 O.D (0.67 mg), the wafers had on average ~40% BCNU remaining in the UC-1 NanoMesh disc. Therefore, preserved NanoMesh after 150 days of implantation will be a plus for even more extensive long-term drug delivery.

Electrospun polymers are capable of withstanding a range of challenging environmental conditions, such as those found in the brain parenchyma, for extended periods of time, without experiencing any significant influence on their structure and integrity, as shown in Fig. 9. The versatility of this platform can be of value in combination with new and more effective chemotherapeutics, by improving the release kinetics of drugs otherwise toxic and decreasing the risk of local and systemic toxicities imputable to burst release. These membranes could improve bioavailability of previously undeliverable drugs and safely achieve local cytotoxic concentrations of potent molecules. The promising release kinetics, safety profile, and layered structure of the NanoMesh membranes also suggest its use for multi-modal therapies. Combination release of multiple drugs with varying mechanisms of action over long periods of time would herald a new era in drug delivery for the treatment of GBM.

## Conclusion

In summary, the results presented here show that long-term release of BCNU can be achieved using multi-layered membranes made of core-sheath fibers, which in turn significantly improved *in vivo* efficacy in rodents bearing 9L gliosarcoma. While in this report a single drug is released from the NanoMesh discs, fiber modifications can be incorporated with two or more drugs in order to provide additional therapeutic value. Current systemic chemotherapy treatments often use combinations of multiple agents to decrease the risk of resistance and provide synergistic effect against tumor cells. Additionally, NanoMesh membranes could incorporate different hydrophobic and hydrophilic compounds on the basis of their efficacy and resistance in treating a specific malignancy, tailoring the specific drugs given in a more personalized manner, and providing alternative and effective opportunities.

## Methods

### Materials

Chemotherapeutic agent, BCNU, was purchased from MilliporeSigma (St. Louis, MO). The core polymer, pCPP-SA, was obtained from Guilford Pharmaceuticals (Baltimore, MD) while the sheath polymer, poly(ε-caprolactone) (PCL, Mn = 80 kDa), was purchased from MilliporeSigma (St. Louis, MO). Solvents such as 2,2,2,-trifluoroethanol (TFE, 99.8% purity), and dichloromethane (DCM) solvents were obtained from Fisher Scientific (Pittsburgh, PA). Female F344 rats were purchased from Charles River Laboratories (Wilmington, MA).

### Sample preparation

All NanoMesh samples (UC-1, UC-2, No Core, No Drug) were produced by using coaxial electrospinning. Core solution was prepared by dissolving 10 wt. % of pCPP-SA polymer and 2 wt. % of BCNU in dichloromethane (DCM, methylene chloride) solvent. Sheath solution was prepared by dissolving 10 wt. % of PCL into the mixture solvent of DCM and TFE in the weight ratio of 6:4. TFE was added to improve electrospinning actions by lowering vapor pressure while maintaining good polymer solubility. DCM is often used as a solvent for BCNU. All solutions are homogenized overnight at 20 RPM using rotating stirrer. Once prepared, each solution was loaded into a syringe and uniformly fed into the corresponding coaxial nozzle outlet by a syringe pump. Core and sheath solutions were fed at 0.6 and 0.8 mL/hr for UC-1 samples, and 0.6 and 1.6 mL/hr for UC-2 samples, respectively. External voltage (~15 kV) was applied across a ~20 cm gap between the coaxial nozzle and the collecting substrate. For “No Core” control samples, the core was formed using DCM solvent only, while “No Drug” controls were produced with the pCPP-SA core solution without BCNU. Membranes with core-sheath fibers encapsulating BCNU in pCPP-SA core and PCL sheath have been successfully produced with different sheath thickness using coaxial electrospinning (See Table 1). Because coaxial electrospinning provides nearly 100% drug encapsulation efficiency, BCNU amounts in Table 1 were calculated considering material/solvent densities, core/sheath flow rates, and weights of NanoMeshes.

**Table 1:** Material composition of NanoMesh discs for rat *in vivo* experiments.

	UC-1		UC-2	
	Disc (mg)	BCNU (mg)	Disc (mg)	BCNU (mg)
Trial 1 (Day 0)	8.29	0.67 (8%)	7.88	0.41 (5.2%)
Trial 2 (Day 5)	10.12	0.80 (7.9%)	8.56	0.44 (5.1%)
Trial 3 (Day 5)	9.93	0.77 (7.8%)	N/A	N/A

### Drug release kinetic measurement

Optical absorption spectrum was used to characterize drug release kinetics in solution using the Thermo Scientific NanoDrop One UV-vis spectrometer. The prepared membrane discs were immersed into 15 mL of PBS buffer solution (pH 7.4). Then, 3 µL of solution was taken at specific time intervals to measure the optical absorption of the solution. Background correction of the absorption spectrum was made with pure PBS buffer solution. At each optical measurement, the maximum absorption intensity at ~245 nm was used to quantify the released amount of BCNU/pCPP-SA core. All measurements were repeated three times at room temperature and a relative humidity of ~40%.

### Intracranial orthotopic rat model

All animals were housed at the Johns Hopkins University animal facility and were given free access to food and Baltimore City water. Animal experiments were approved by the Johns Hopkins University Animal Care and Use Committee (ACUC) and followed its guidelines throughout the length of the study. For *in vivo* experiments the 9L gliosarcoma model was used, and microscopic tumor pieces were implanted intracranially in 94 animals (n = 31 for the first experiment, 47 for the second and 16 for the third). The 9L gliosarcoma was originally obtained from the Brain Tumor Research Center, San Francisco, CA, USA. For tumor piece implantation, 9L tumor pieces measuring 2 mm^3^ were passaged in the flank of F344 rats every 3 to 4 weeks. For intracranial implantation, the 9L gliosarcoma tumor was surgically excised from the carrier animal using sterile technique, cut into 1-mm^3^ pieces (approximately 5 × 10^5^ cells), placed in sterile 0.9% NaCl solution and kept on ice at 4 °C. This tumor piece method is more aggressive than cellular injection and represents the clinical condition more accurately. The animals that received intracranial tumors were anesthetized with an intraperitoneal (IP) injection of a 3 mL/kg solution containing ketamine hydrochloride 75 mg/ml (Ketathesia, Butler Animal Health Supply; Dublin, OH), xylazine 7.5 mg/mL (Lloyd Laboratories; Shenandoah, Iowa) and 14.25% ethyl alcohol in 0.9% NaCl. The head was shaved, prepared with Prepodyne solution (West Penetone, Montreal, Canada) and incised along the midline, exposing the coronal and sagittal sutures. A 3 mm burr hole was drilled in the left parietal bone, 3 mm posterior from the coronal suture and 3 mm lateral from the sagittal suture, posterolateral to bregma. A small portion of cortical and subcortical matter was removed by aspiration under surgical microscope magnification, and the 2 mm^3^ 9L tumor piece was implanted in the left hemisphere. The skin was closed with surgical clips and animals were returned to their cages and allowed to awaken. Access to food and water was unrestricted and all the animals were monitored post-operatively and for the entire length of the study for signs of toxicity, neurologic compromise, bleeding and failure to thrive.

### Intracranial wafer implantation

Animals treated on Day 0 were randomized into four groups (untreated control, empty control membrane, UC-1, and UC-2; n = 31) and administered intracranial NanoMesh discs immediately following tumor placement. Discs were implanted vertically in the same pocket with the tumor.

Two additional efficacy studies were carried out with treatment given 5 days after tumor insertion (Trial #2 groups included: untreated control, empty control wafer, UC-1 and UC-2, n = 47. Trial #3 groups included: untreated control and UC-1, n = 16). Animals were randomized and anesthetized following the previously described procedure. NanoMesh discs were positioned vertically inside the originally burred tumor pocket and the wound was closed with surgical clips.

Throughout each of these studies, animals were closely monitored for signs of systemic illness (lethargy, failure to thrive), neurological decline (seizures, paralysis, gait abnormalities) and brain tumor growth (lethargy, huddled behavior, lack of grooming and eating). Any animals found with any indications were euthanized according to Animal Care and Use Committee (ACUC)-approved protocols and their brains were harvested and fixed for histological analysis. Those that survived until Day 150 were deemed long-term survivors (LTS) and perfused with 4% paraformaldehyde (Sigma Aldrich, St. Louis, MO) to achieve brain fixation. Tumor growth was assessed by necropsy and histological examination.

### Histological analysis

Brains were collected immediately after euthanasia, preserved in 10% formalin (Sigma Aldrich, St. Louis, MO), and embedded in paraffin. Brains of healthy animals intentionally euthanized at prefixed time points were perfused with 4% paraformaldehyde (Sigma Aldrich, St. Louis, MO) and switched to 10% formalin after 24 hours for long-term preservation. All samples were paraffin-embedded by the Johns Hopkins University Histopathology Core and stained in hematoxylin-eosin (Sigma Aldrich, St. Louis, MO). Brains were evaluated for tumor growth, wafer degradation, and tissue damage. For immunohistochemistry, representative slides of polymeric wafer and tumor were prepared and stained for an astrocytic marker, GFAP (1:100; Abcam, Cambridge, UK), and NeuN (1:100; Abcam, Cambridge, UK), for viable neurons. Brains were deparaffinized and antigens retrieved and blocked with Avidin Biotin System Block (Vector Laboratories Inc, Burlingame, CA, USA) and goat or donkey serum. Primary and secondary antibodies were prepared with either goat or donkey serum. DAB solution was prepared and applied, and stain evaluated under the microscope. Slides were then stained in hematoxylin (Sigma Aldrich, St. Louis, MO), washed, dehydrated and mounted on coverslips with Cytoseal™ XYL (Richard Allan Scientific™, San Diego, CA, USA). Images were taken with a Zeiss^®^ Axiovert Microscope (Carl Zeiss, Oberkochen, Germany) at different magnifications.

### Electron microscopy analysis

Because of insulative nature of polymeric NanoMeshes, all samples were coated with conductive thin Au layers of using Denton Desk II mini-sputtering system. Fiber morphologies were obtained using the benchtop mini-SEM (SciXR SX-3000) with the acceleration voltage of 20 kV. For the cross-sectional observation of NanoMesh samples, they were first frozen in −80° C freezer, then immediately cut into slices using a X-Acto knife.

### Statistical analysis

All statistical analyses were carried out using GraphPad Prism^®^ (Version 8.1, GraphPad Software, San Diego, CA) and statistical significance was set to a P value of 0.05. One-way ANOVA with Bonferroni and Tukey post-tests or a non-parametric Kruskal-Wallis test were performed, depending on the distribution of the data. In survival studies, survival was analyzed using the Kaplan-Meier estimator, and statistical significance was established using log-rank analysis.

## Supplementary information

Supplementary information

## References

[1] Chen Shixuan, Boda Sunil Kumar, Batra Surinder K., Li Xiaoran, Xie Jingwei (2017). Emerging Roles of Electrospun Nanofibers in Cancer Research. Advanced Healthcare Materials.

[2] Louis DN (2016). The 2016 World Health Organization Classification of Tumors of the Central Nervous System: a summary. Acta Neuropathologica.

[3] Omuro A, DeAngelis LM (2013). Glioblastoma and Other Malignant Gliomas: A Clinical Review. JAMA.

[4] Stupp R (2005). Radiotherapy plus Concomitant and Adjuvant Temozolomide for Glioblastoma. N. Engl. J. Med..

[5] Daneman Richard, Prat Alexandre (2015). The Blood–Brain Barrier. Cold Spring Harbor Perspectives in Biology.

[6] Brem H (1994). Biodegradable polymers for controlled delivery of chemotherapy with and without radiation therapy in the monkey brain. Journal of Neurosurgery.

[7] McGirt MJ (2009). Gliadel (BCNU) wafer plus concomitant temozolomide therapy after primary resection of glioblastoma multiforme. Journal of neurosurgery.

[8] Brem H (1991). Interstitial chemotherapy with drug polymer implants for the treatment of recurrent gliomas. Journal of Neurosurgery.

[9] Brem H (1995). Placebo-controlled trial of safety and efficacy of intraoperative controlled delivery by biodegradable polymers of chemotherapy for recurrent gliomas. The Lancet.

[10] Menei P (2010). Biodegradable Carmustine Wafers (Gliadel) Alone or in Combination with Chemoradiotherapy: The French Experience. Annals of Surgical Oncology.

[11] Duntze J (2013). Implanted Carmustine Wafers Followed by Concomitant Radiochemotherapy to Treat Newly Diagnosed Malignant Gliomas: Prospective, Observational, Multicenter Study on 92 Cases. Annals of Surgical Oncology.

[12] Chowdhary SA, Ryken T, Newton HB (2015). Survival outcomes and safety of carmustine wafers in the treatment of high-grade gliomas: a meta-analysis. Journal of Neuro-Oncology.

[13] Xue J, Xie J, Liu W, Xia Y (2017). Electrospun Nanofibers: New Concepts, Materials, and Applications. Accounts of Chemical Research.

[14] Moghe AK, Gupta BS (2008). Co-axial Electrospinning for Nanofiber Structures: Preparation and Applications. Polym. Rev..

[15] Xie J, Wang C-H (2006). Electrospun Micro- and Nanofibers for Sustained Delivery of Paclitaxel to Treat C6 Glioma *in Vitro*. Pharmaceutical Research.

[16] Xu X (2006). BCNU-loaded PEG–PLLA ultrafine fibers and their *in vitro* antitumor activity against Glioma C6 cells. Journal of Controlled Release.

[17] Huang H-H, He C-L, Wang H-S, Mo X-M (2009). Preparation of core-shell biodegradable microfibers for long-term drug delivery. Journal of Biomedical Materials Research Part A.

[18] Tseng Y-Y, Liao J-Y, Chen W-A, Kao Y-C, Liu S-J (2013). Sustainable release of carmustine from biodegradable poly[(d,l)-lactide-co-glycolide] nanofibrous membranes in the cerebral cavity: *in vitro* and *in vivo* studies. Expert Opinion on Drug Delivery.

[19] Iqbal S, Rashid MH, Arbab AS, Khan M (2017). Encapsulation of Anticancer Drugs (5-Fluorouracil and Paclitaxel) into Polycaprolactone (PCL) Nanofibers and *In Vitro* Testing for Sustained and Targeted Therapy. Journal of Biomedical Nanotechnology.

[20] Barth RF, Kaur B (2009). Rat brain tumor models in experimental neuro-oncology: the C6, 9L, T9, RG2, F98, BT4C, RT-2 and CNS-1 gliomas. Journal of Neuro-Oncology.

[21] Han D, Boyce ST, Steckl AJ (2008). Versatile core-sheath biofibers using coaxial electrospinning. Mater. Res. Soc. Symp. Proc..

[22] Wang N (2014). Electrospun polyurethane-core and gelatin-shell coaxial fibre coatings for miniature implantable biosensors. Biofabrication.

[23] Wu D, Han D, Steckl AJ (2010). Immunoassay on free-standing electrospun membranes. ACS Applied Materials and Interfaces.

[24] Shapira-Furman T (2019). Biodegradable wafers releasing Temozolomide and Carmustine for the treatment of brain cancer. Journal of Controlled Release.

[25] Zhang C (2017). Convection enhanced delivery of cisplatin-loaded brain penetrating nanoparticles cures malignant glioma in rats. Journal of Controlled Release.

[26] Smith SJ (2019). Overall survival in malignant glioma is significantly prolonged by neurosurgical delivery of etoposide and temozolomide from a thermo-responsive biodegradable paste. Clinical Cancer Research, clincanres..

[27] Mangraviti A (2015). Polymeric Nanoparticles for Nonviral Gene Therapy Extend Brain Tumor Survival *in Vivo*. ACS Nano.

[28] Mathios D (2016). Anti–PD-1 antitumor immunity is enhanced by local and abrogated by systemic chemotherapy in GBM. Science Translational Medicine.

