# Supplementary material for: Multi-layered core-sheath fiber membranes for controlled drug release in the local treatment of brain tumor
Source: Sci Rep. 2019 Nov 29;9:17936. doi: 10.1038/s41598-019-54283-y (PMC6884550; doi:10.1038/s41598-019-54283-y)
Supplement: Supplementary file 1 — Supplementary information [file 41598_2019_54283_MOESM1_ESM.docx]

**Multi-layered core-sheath fiber membranes for controlled drug release in the local treatment of brain tumor**

# Daewoo Han1, Riccardo Serra2, Noah Gorelick2, Umailla Fatima2, Charles G. Eberhart^3^, Henry Brem2,4, Betty Tyler2, and Andrew J. Steckl1,*

1Nanoelectronics Laboratory, Electrical Engineering and Computer Science, University of Cincinnati, OH USA

2Department of Neurosurgery, Johns Hopkins University School of Medicine, Baltimore, MD USA

3Departments of Pathology, Oncology, and Ophthalmology, Johns Hopkins University School of Medicine, Baltimore, MD USA

4Departments of Biomedical Engineering, Oncology, and Ophthalmology, Johns Hopkins University School of Medicine, Baltimore, MD USA

*corresponding Author: Andrew Steckl: [a.steckl@uc.edu](mailto:a.steckl@uc.edu)


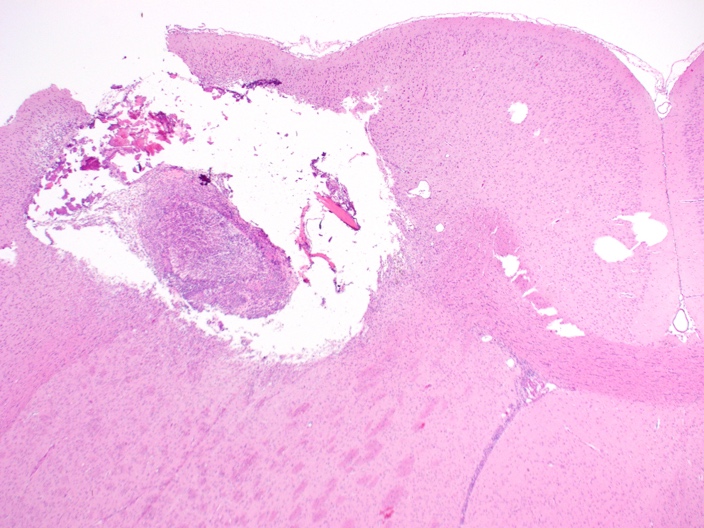

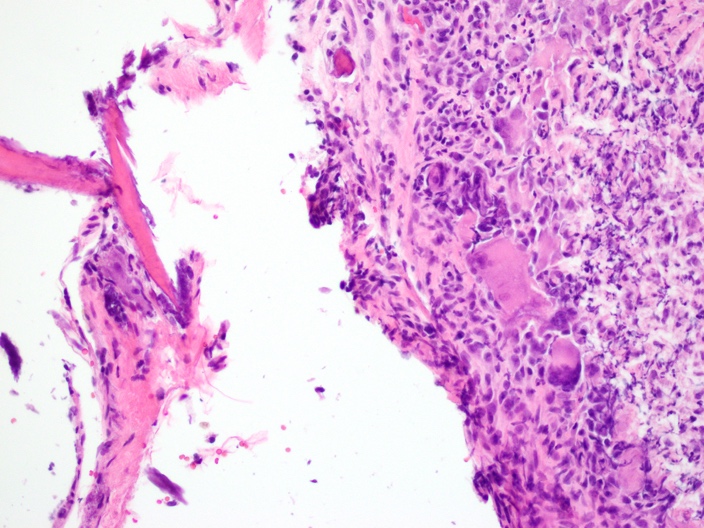


# *

# *

**(a)**

**(b)**

# Figure S1. Histological reaction of rat brain parenchyma to No Drug control disc. (a) No Drug control disc remnants found throughout the implantation cavity of a rat brain two weeks following implantation. Healthy brain parenchyma surrounding the implantation cavity shows slight evidence of reactive gliosis, hemorrhage, inflammation, and foreign body giant cells consistent with operative changes (Hematoxylin and Eosin staining at 20× magnification); (b) A higher magnification image from within the same section (200 ×) shows fragments of No Drug control disc (*) and its surrounding parenchyma with evidence of reactive, multinucleated foreign body giant cells (arrow).


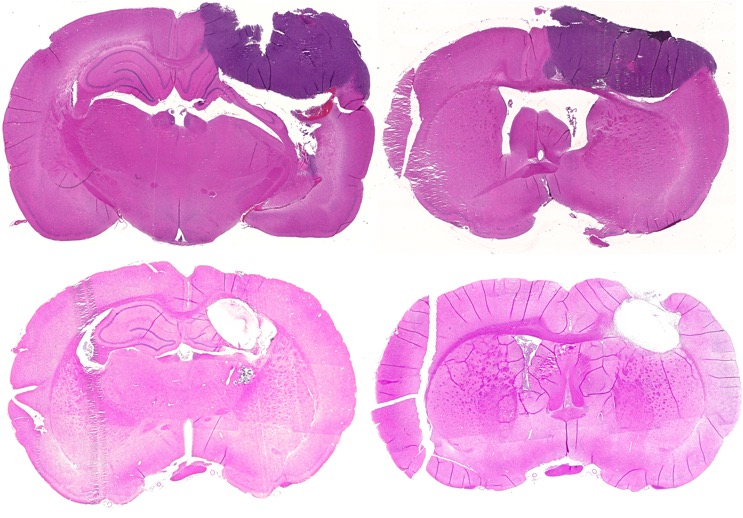


**Figure S2.** Trial 1 Histology (Day 0 Treatment). Brains from No Core NanoMesh (day 14) control groups. Both rats succumbed to intracranially implanted 9L gliosarcoma, with brains showing gross tumor and microscopic evidence of diffuse infiltrating malignant cells in the left hemispheres. (5× magnification, stained with hematoxylin & eosin).


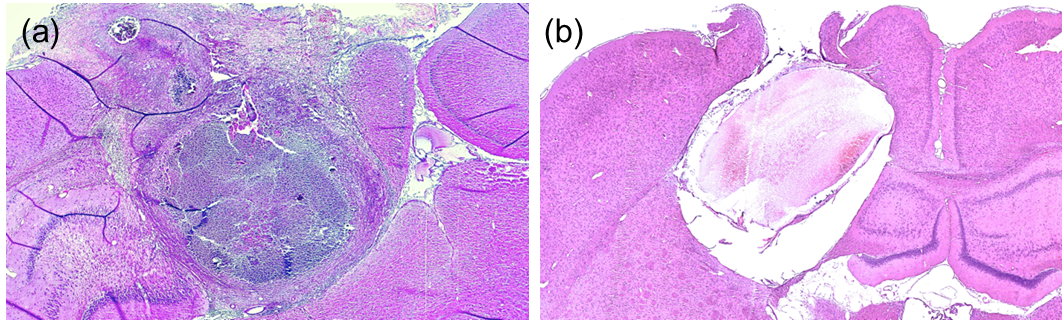


**Figure S3.** Histological sections from rats intracranially implanted with 9L tumor and UC-1 fiber membranes on Day 0 from Trial 1: (a) asymptomatic animal euthanized on Day 40 shows a diffuse inflammatory response surrounding the implant; (b) asymptomatic animal euthanized on Day 150 shows a marked decrease in the inflammatory response and presents a mild degree of cellular infiltration, confirming no signs of toxicity from the implants. Only a modest degradation of the original discs was observed in these samples. (10**×** magnification, stained with hematoxylin & eosin).


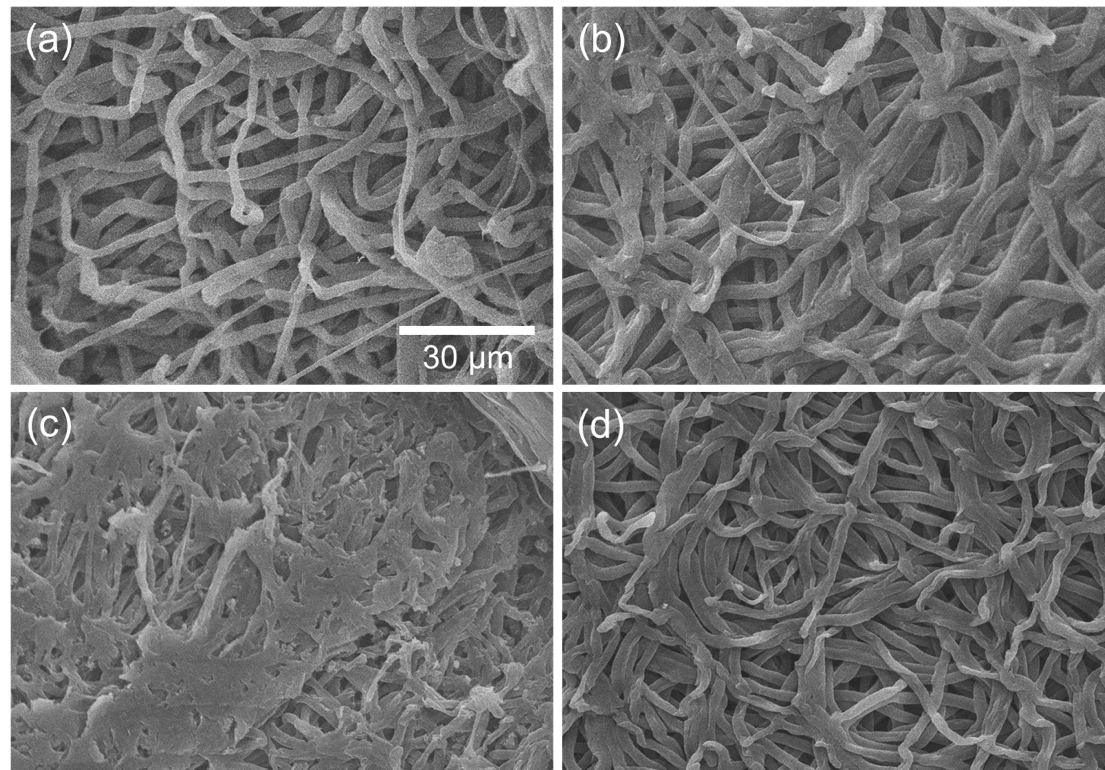


**Figure S4.** SEM images of the middle layer of post-implantation NanoMesh discs from Trial 2 samples: (a) “No Core” (15 days post-implantation); (b) “No Drug” (20 days post-implantation); (c) UC-1 (32 days post-implantation); (d) UC-2 (35 days post-implantation).
